# Assessing the impact of liver fat fraction on portal hemodynamics in nonalcoholic fatty liver disease using MRI proton density fat fraction and MRI 4D Flow

**DOI:** 10.3389/fmed.2025.1620649

**Published:** 2025-08-07

**Authors:** Li-Zhen Wang, Wen-Qiang Li, Yao Li, Xiao-Yan Li, Shuai Ju

**Affiliations:** ^1^Department of Nephrology, Jinshan Hospital of Fudan University, Shanghai, China; ^2^Department of Vascular and Wound Center, Jinshan Hospital of Fudan University, Shanghai, China

**Keywords:** nonalcoholic fatty liver disease, liver fat fraction, portal hemodynamics, MRI, MRI 4D Flow, hepatic steatosis, vascular dynamics

## Abstract

**Background:**

Nonalcoholic fatty liver disease (NAFLD) is a prevalent condition with significant implications for liver and cardiovascular health. Alterations in portal hemodynamics due to hepatic steatosis remain poorly understood.

**Aim:**

This study aims to explore the correlation between liver fat fraction (FF) and portal hemodynamics in NAFLD patients.

**Methods:**

A retrospective observational study was conducted involving 125 clinical suspected NAFLD patients. Liver FF was measured using MRI proton density fat fraction (PDFF). MRI 4D Flow was used to assess portal hemodynamic parameters, including flow velocity, flow volume, and portal area. Statistical analyses examined the relationships between liver FF and hemodynamic parameters.

**Results:**

Liver FF was negatively associated with portal peak flow velocity (*r* = −0.33) and portal mean flow velocity (*r* = −0.49), but was positively correlated with portal area (*r* = 0.39). No correlation was found in liver FF and portal flow volume (*p* = 0.114). Portal peak velocity demonstrated AUCs of 0.69 (95% CI: 0.57–0.82) for differentiating G0 from G1-3, 0.70 (95% CI: 0.60–0.79) for G0-1 versus G2-3, and 0.57 (95% CI: 0.44–0.69) for G0-2 versus G3. Portal mean velocity demonstrated AUCs of 0.84 (95% CI: 0.76–0.92) for differentiating G0 from G1-3, 0.78 (95% CI: 0.69–0.86) for G0-1 versus G2-3, and 0.70 (95% CI: 0.60–0.79) for G0-2 versus G3. Portal area demonstrated AUCs of 0.79 (95% CI: 0.70–0.78) for G0 versus G1-3, 0.78 (95% CI: 0.48–0.92) for G0-1 versus G2-3, and 0.84 (95% CI: 0.76–0.92) for G0-2 versus G3.

**Conclusion:**

Liver FF is a significant determinant of portal hemodynamics in NAFLD patients. These findings underscore the potential of integrating liver FF and portal hemodynamic assessments into clinical practice for detection and management of NAFLD progression.

## Introduction

Nonalcoholic fatty liver disease (NAFLD) is a growing global health concern, which affects approximately 17–46% of the adult population worldwide (1). NAFLD includes a spectrum of liver conditions ranging from simple steatosis to steatohepatitis, which may progress to liver fibrosis, cirrhosis, and hepatocellular carcinoma (2). NAFLD has significant implications for cardiovascular and metabolic health (3). Thus, it is important for clinical practice in detection and management of NAFLD progression.

Changes in hepatic structure and function, such as fibrosis and cirrhosis, can lead to portal hypertension with significantly altered portal pressure and flow velocity (4, 5). Previous studies showed that subclinical portal hypertension was also repeatedly detected in patients with NAFLD (6). These findings suggest that liver fat accumulation alters the mechanical and vascular environment of the liver, contributing to increased portal pressure and alterations in portal hemodynamics (7). By using ultrasound, recent studies highlighted significant correlations between hepatic steatosis and portal hemodynamics in NAFLD patients. Reductions in portal flow velocity and increases in portal diameter have been observed in patients with advanced hepatic steatosis (8). These insights emphasize the importance of assessing both metabolic and portal hemodynamic changes in understanding the progression of NAFLD. Understanding these changes is critical for identifying noninvasive diagnostic and prognostic markers for NAFLD progression.

Magnetic resonance imaging (MRI) provides a promising platform for assessing hepatic fat content and portal hemodynamics, simultaneously and noninvasively. MRI proton density fat fraction (MRI PDFF) and MRI 4D Flow allow for detailed quantification of liver fat fraction (FF) and portal hemodynamics, respectively (9, 10). Previous study shows that MRI PDFF is better in quantifying FF than ultrasound even than biopsy (11). MRI 4D Flow is also successfully compared to ultrasound in monitoring portal hemodynamic changes (12). Using MRI for examination of liver FF and portal hemodynamics enables researchers to investigate correlations between liver steatosis and portal hemodynamics with greater precision and reliability. However, the diagnostic performance of MRI 4D Flow in NAFLD staging is not well studied.

We proposed that increased hepatic fat accumulation leads to measurable changes in portal hemodynamic changes. Thus, the aim of this study is to provide a understanding of the portal hemodynamics changes in hepatic steatosis and to explore the potential utility of MRI 4D Flow as diagnostic indicators for NAFLD staging.

## Methods

### Ethnic and study design

This retrospective observational study was approved by Institutional ethical approval (No. JIEC 2025-S35). The study adhered to the Declaration of Helsinki guidelines. All participants provided written informed consent after a thorough explanation of the study’s objectives and procedures.

### Patient enrollment

From October 2, 2020, to April 2, 2025, participants were enrolled through the local PACS system. Screening procedures involved detailed medical history reviews, physical examinations, and preliminary imaging assessments of NAFLD, such as abdominal ultrasounds and MRI.

### Inclusion criteria

The study included adults aged 18–90 years, who were suspected as NAFLD clinically. All cases should be comply with study requirements, including MRI scans of both MRI PDFF and MRI 4D Flow.

### Exclusion criteria

Exclusion criteria involved alcohol consumption exceeding 40 g/day for men or 20 g/day for women. Participants were also excluded if they were diagnosed with other chronic liver diseases, such as viral hepatitis, autoimmune hepatitis, or Wilson’s disease. Additionally, participants with incomplete imaging data or poor-quality MRI scans were excluded from the study. Cases with focal fatty liver disease were also excluded, which may affect the parameters of portal blood flow.

### Clinical laboratory tests

The clinical laboratory tests was retrieved form the local HIS system. Blood test within 30 days before the MR examination were collected. Blood samples were collected after a 12-h overnight fast. Blood pressure was recorded. Plasma glucose levels were assessed using enzymatic methods, while total cholesterol, triglycerides, high-density lipoprotein (HDL), and low-density lipoprotein (LDL) were measured using enzymatic colorimetric assays. Liver enzyme levels, including aspartate aminotransferase (AST) and alanine aminotransferase (ALT), were quantified using automated spectrophotometric techniques. Total bilirubin (TB) levels were determined through the diazo reaction method.

### MRI scanning and parameters for liver FF measurement

All participants underwent MRI scans on a 3.0 T scanner (uMR 780, United Imaging, China) equipped with a body phased-array coil. The liver FF was quantified using the FACT sequence, which calculates MRI PDFF. Imaging parameters included a repetition time of 10.8 ms, echo times ranging from 1.72 ms to 9.34 ms, a slice thickness of 6 mm, and a field of view of 380 × 380 mm. Regions of interest (ROIs) were placed in both hepatic lobes, avoiding major vessels and bile ducts, to compute the mean liver FF as the average of these measurements. Four ROIs, each approximately 0.3 cm^2^ in size, were randomly placed in the left and right hepatic lobes. The average FF values for the left and right lobes were used to determine the overall liver FF. Based on FF values, liver steatosis was classified into four grades: Grade 0 (S0, FF 0–5%), Grade 1 (S1, FF 5–15%), Grade 2 (S2, FF 15–19%), and Grade 3 (S3, FF > 19%). Grades 2 and above were considered indicative of fatty liver disease (9).

### MRI scanning and parameters for MRI 4D Flow in portal hemodynamics

MRI 4D Flow was conducted using a breath-hold protocol to reduce motion artifacts, with parameters including a repetition time of 44.16 ms, echo time of 3.25 ms, and slice thickness of 6 mm. The acquisition time was approximately 15 min. Axial, coronal, and sagittal planes were reconstructed to locate the portal vein. ROIs were manually placed on the cross-sections of the portal vein trunk, left portal vein, and right portal vein at approximately 1 cm from the bifurcation. The size of the ROIs was matched to the vessel diameter. Measurements of peak flow velocity, mean flow velocity, portal flow volume, and portal diameter were recorded. The final result for each measurement was calculated as the mean of two separate measurements.

### Statistical analysis

Data analyses were performed using R software (Version 4.5.0). Continuous variables were expressed as mean ± standard deviation, while categorical variables were presented as frequencies and percentages. For intergroup comparisons, Student’s t-test was used for parametric data, and Kruskal-Wallis tests were applied for nonparametric data. Correlation analyses between liver FF and portal hemodynamic parameters were conducted using Pearson’s or Spearman’s correlation methods, depending on data distribution. Receiver operating characteristic (ROC) curves were generated to evaluate the diagnostic performance of MRI 4D Flow parameters for detecting varying degrees of liver fat contents. The optimal cutoff values were determined by analyzing sensitivity, specificity, positive predictive value (PPV), and negative predictive value (NPV) at different thresholds. Statistical significance was defined as *p* < 0.05.

## Results

### Patient enrollment

**Figure 1:**
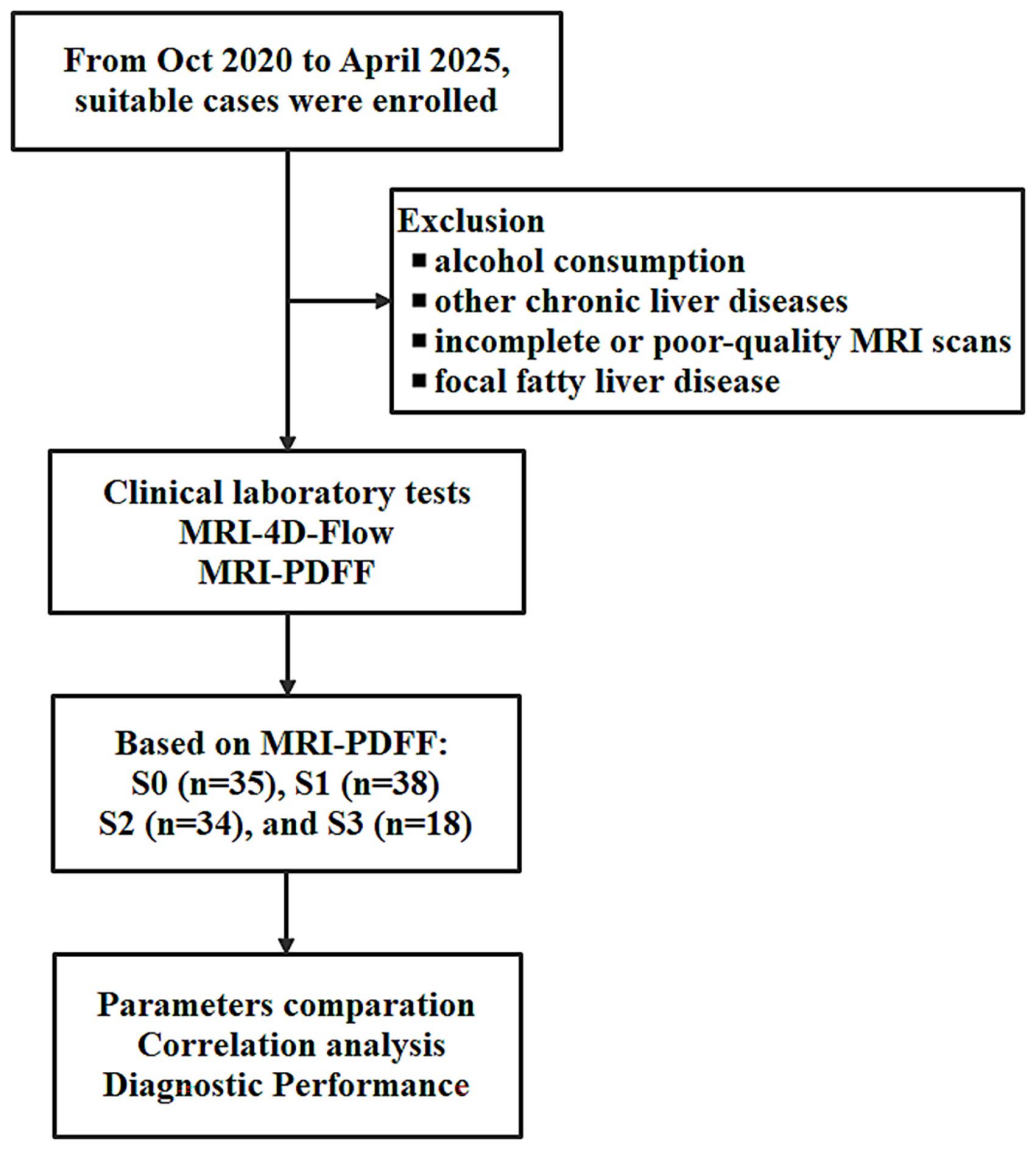
The work flow of this study for non-alcoholic fatty liver disease (NAFLD) assessment using MRI 4D Flow and MRI proton density fat fraction (MRI PDFF).

The workflow of this study is illustrated in Figure 1. Finally, 125 participants who were divided into four groups based on MRI PDFF: G0 (*n* = 35), G1 (*n* = 38), G2 (*n* = 34), and G3 (*n* = 18). For G0 cases, the age was 56 ± 15.1 (27–86). For G1 cases, the age was 52 ± 14.6 (26–74). For G2 cases, the age was 52 ± 14.6 (34–87). For G3 cases, the age was 57 ± 8.4 (43–77). Female cases representation declined with increasing steatosis severity (57.1% in G0, 42.1% in G1, 32.4% in G2, and 33.3% in G3). Age and gender did not differ significantly across the groups. Table 1 summaries the clinical characteristics and laboratory tests for all groups.

**Table 1:** The clinical characteristics and laboratory tests for all groups.

Variable	G0	G1	G2	G3	*p*-value#	*p*-value$	*p*-value*
	(*N* = 35)	(*N* = 38)	(*N* = 34)	(*N* = 18)			
Gender					0.060	0.095	0.560
Female	20 (57.1%)	16 (42.1%)	11 (32.4%)	6 (33.3%)			
Male	15 (42.9%)	22 (57.9%)	23 (67.6%)	12 (66.7%)			
Age	56 ± 15.1	52 ± 14.6	52 ± 14.5	57 ± 8.4	0.219	0.775	0.152
BP-S (mmHg)	145 ± 10.3	150 ± 10.4	156 ± 8.5	154 ± 18.6	0.001	<0.001	0.413
BP-D (mmHg)	83 ± 7.2	88 ± 10.5	93 ± 10.2	92 ± 7.0	<0.001	<0.001	0.050
Glucose (mg/dL)	6.1 ± 1.08	7.2 ± 1.46	7.1 ± 1.46	7.2 ± 2.05	<0.001	0.013	0.469
Cholesterol (mg/dL)	5.5 ± 1.09	5.92 ± 0.452	6.5 ± 0.869	7.3 ± 1.39	<0.001	<0.001	0.001
Triglyceride (mg/dL)	1.5 ± 0.46	1.9 ± 0.22	2.4 ± 1.34	3.5 ± 1.76	<0.001	<0.001	0.002
HDL (mg/dL)	1.4 ± 0.34	1.5 ± 0.522	1.1 ± 0.42	1.2 ± 0.27	0.037	<0.001	0.017
LDL (mg/dL)	3.4 ± 0.72	3.7 ± 0.42	4.1 ± 0.73	4.1 ± 0.83	<0.001	<0.001	0.078
AST (U/L)	25 ± 8.2	30 ± 10.3	42 ± 19.3	55 ± 21.5	<0.001	<0.001	<0.001
ALT (U/L)	23 ± 15.0	31 ± 10.7	63 ± 29.5	55 ± 5.1	<0.001	<0.001	<0.001
TB (mg/dL)	2.8 ± 1.0	2.7 ± 0.9	3.4 ± 1.0	4.1 ± 1.3	0.062	<0.001	0.003
Mean FF	3.7 ± 0.58	7.2 ± 1.34	16.9 ± 1.38	24.6 ± 5.93	<0.001	<0.001	<0.001
Peak velocity-P (cm/s)	20.5 ± 4.38	18.6 ± 3.89	16.6 ± 1.20	17.4 ± 2.16	0.001	<0.001	0.055
Mean velocity-P (cm/s)	15.9 ± 2.71	13.3 ± 3.20	11.8 ± 1.54	11.6 ± 1.38	<0.001	<0.001	<0.001
Flow-P (mL/s)	9.8 ± 2.72	11.3 ± 4.59	11.2 ± 3.19	11.6 ± 1.59	0.081	0.220	0.110
Area-P (cm^2^)	0.6 ± 0.24	0.8 ± 0.29	0.9 ± 0.25	1.0 ± 0.10	<0.001	<0.001	<0.001
Peak velocity-L (cm/s)	11.2 ± 2.69	12.1 ± 2.39	11.6 ± 3.68	11.5 ± 3.97	0.288	0.890	0.917
Mean velocity-L (cm/s)	7.7 ± 2.82	9.1 ± 1.82	8.3 ± 2.61	7.6 ± 3.04	0.127	0.472	0.295
Flow-L (mL/s)	2.7 ± 1.32	3.8 ± 2.57	3.5 ± 2.10	3.0 ± 0.76	0.045	0.540	0.188
Area-L (cm^2^)	0.3 ± 0.16	0.4 ± 0.28	0.4 ± 0.35	0.3 ± 0.20	0.285	0.817	0.129
Peak velocity-R (cm/s)	15.6 ± 4.53	15.2 ± 3.51	15.8 ± 1.63	15.5 ± 3.52	0.910	0.606	0.967
Mean velocity-R (cm/s)	9.9 ± 4.21	9.6 ± 2.50	10.9 ± 1.61	10.9 ± 3.90	0.568	0.063	0.461
Flow-R (mL/s)	5.5 ± 2.36	6.4 ± 3.32	7.1 ± 2.20	5.7 ± 2.08	0.041	0.173	0.285
Area-R (cm^2^)	0.5 ± 0.21	0.6 ± 0.32	0.6 ± 0.24	0.6 ± 0.25	0.102	0.661	0.527

ALT, Alanine Aminotransferase; AST, Aspartate Aminotransferase; BP-D, Blood Pressure Diastolic; BP-S, Blood Pressure Systolic; FF, Fat Fraction; HDL, High-Density Lipoprotein; L, Left Portal Vein; LDL, Low-Density Lipoprotein; P, Portal Vein; R, Right Portal Vein; TB, Total Bilirubin.

#, G0 verses G1-3, $, G0-1 verses G2-3; *, G0-2 verses G3.

### Clinical laboratory tests

Compared to the G0 cases, NAFLD cases (G1-G3) exhibited elevated cholesterol (*p* < 0.001, *p* < 0.001, *p* = 0.001), triglyceride (*p* < 0.001, *p* < 0.001, *p* = 0.002), LDL (*p* = 0.037, *p* < 0.001, *p* = 0.017), but decreased HDL (*p* < 0.001, p < 0.001, *p* = 0.078) with increasing steatosis severity (Table 1). Elevated diastolic blood pressure (BP, *p* < 0.001, *p* < 0.001, *p* = 0.050), ALT (*p* < 0.001, *p* < 0.001, *p* < 0.001), AST (*p* < 0.001, *p* < 0.001, *p* < 0.001), and TB (*p* = 0.062, *p* < 0.001, *p* = 0.003) were found in G1-G3 cases comparing to the G0 cases. Blood glucose (*p* < 0.001 and *p* = 0.013) and systolic BP (*p* = 0.001 and *p* < 0.001) were elevated in G1 and G2 cases.

### MRI liver FF measurement

**Figure 2:**
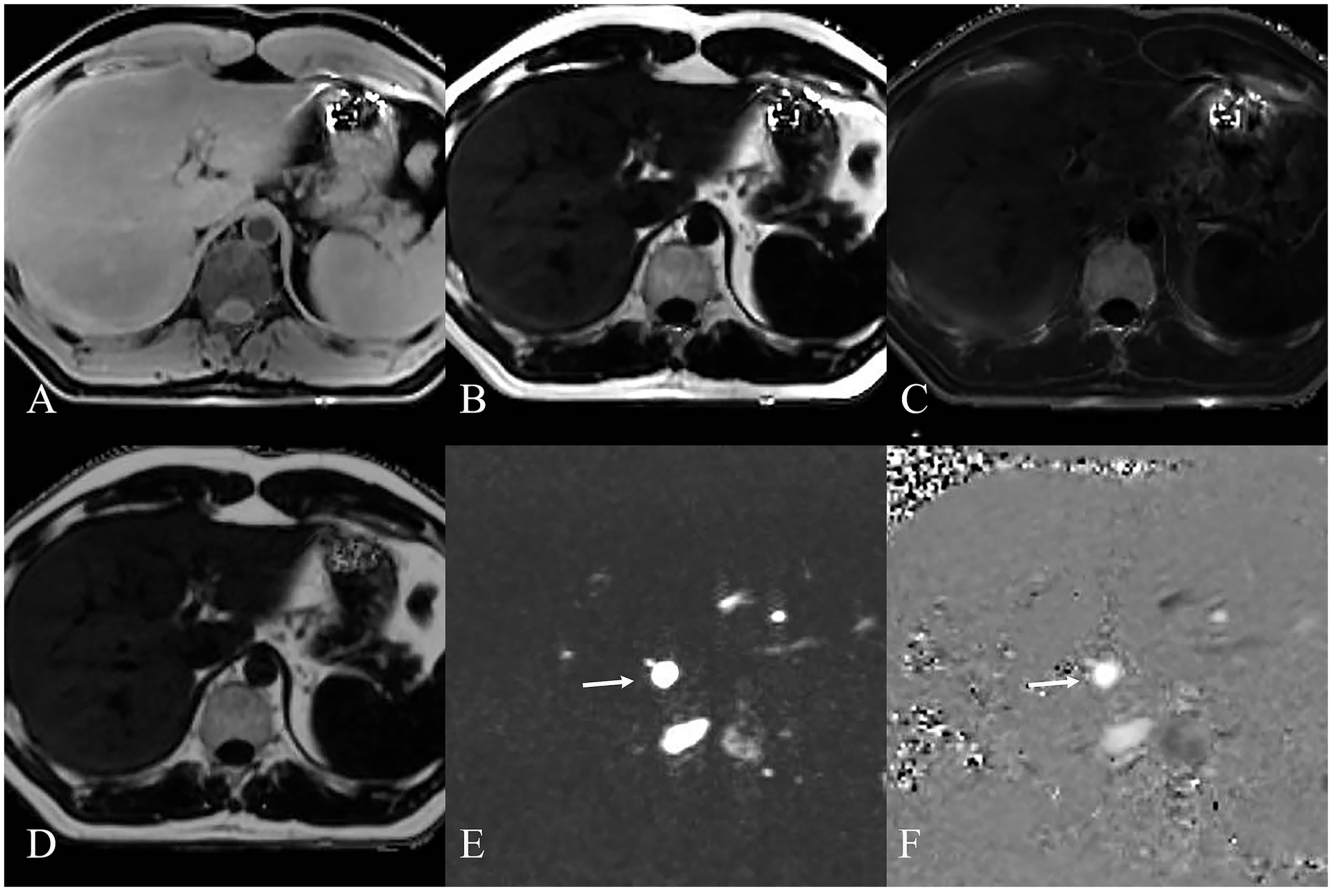
MRI images displaying various sequences used for MRI PDFF **(A–D)** and MRI 4D Flow **(E,F)** measurement of NAFLD. **(A)** Water image shows the water content in the liver. **(B)** Fat image shows the fat distribution in the liver. **(C)** R2* map shows the liver relaxation rate. **(D)** Fat fraction map shows the liver fat content quantifying. MRI 4D Flow magnitude image **(E)** and phase difference image **(F)** show the hepatic hemodynamics with flow visualization in the portal vein (white arrow).

MRI PDFF (demonstrated in Figure 2) confirmed significant higher liver FF with increasing steatosis severity. The mean liver FF was 3.7 ± 0.58 for G0 cases, 7.2 ± 1.34 for G1 cases, 16.9 ± 1.38 for G2 cases, and 24.6 ± 5.93 for G3 cases, respectively.

### MRI 4D Flow in portal hemodynamics

MRI 4D Flow (demonstrated in Figure 2) indicated significantly reduced portal peak flow velocity (*p* < 0.001, *p* < 0.001, *p* = 0.055), portal mean flow velocity (*p* < 0.001, *p* < 0.001, *p* < 0.001) and portal area (*p* < 0.001, *p* < 0.001, *p* < 0.001) with increasing steatosis severity (Table 1). Slight increased portal flow volume were found in G1-G3 cases, but without statistical significance (*p* = 0.081, *p* = 0.220, *p* = 0.110).

No significant difference was shown in most hepatic hemodynamic parameters in the right and left branches of the portal vein. Blood flow volume was only found significantly increased in right (*p* = 0.041) and left (*p* = 0.045) branches of the portal vein in G1 cases (comparing with G0 cases).

### Statistical correlations

**Figure 3:**
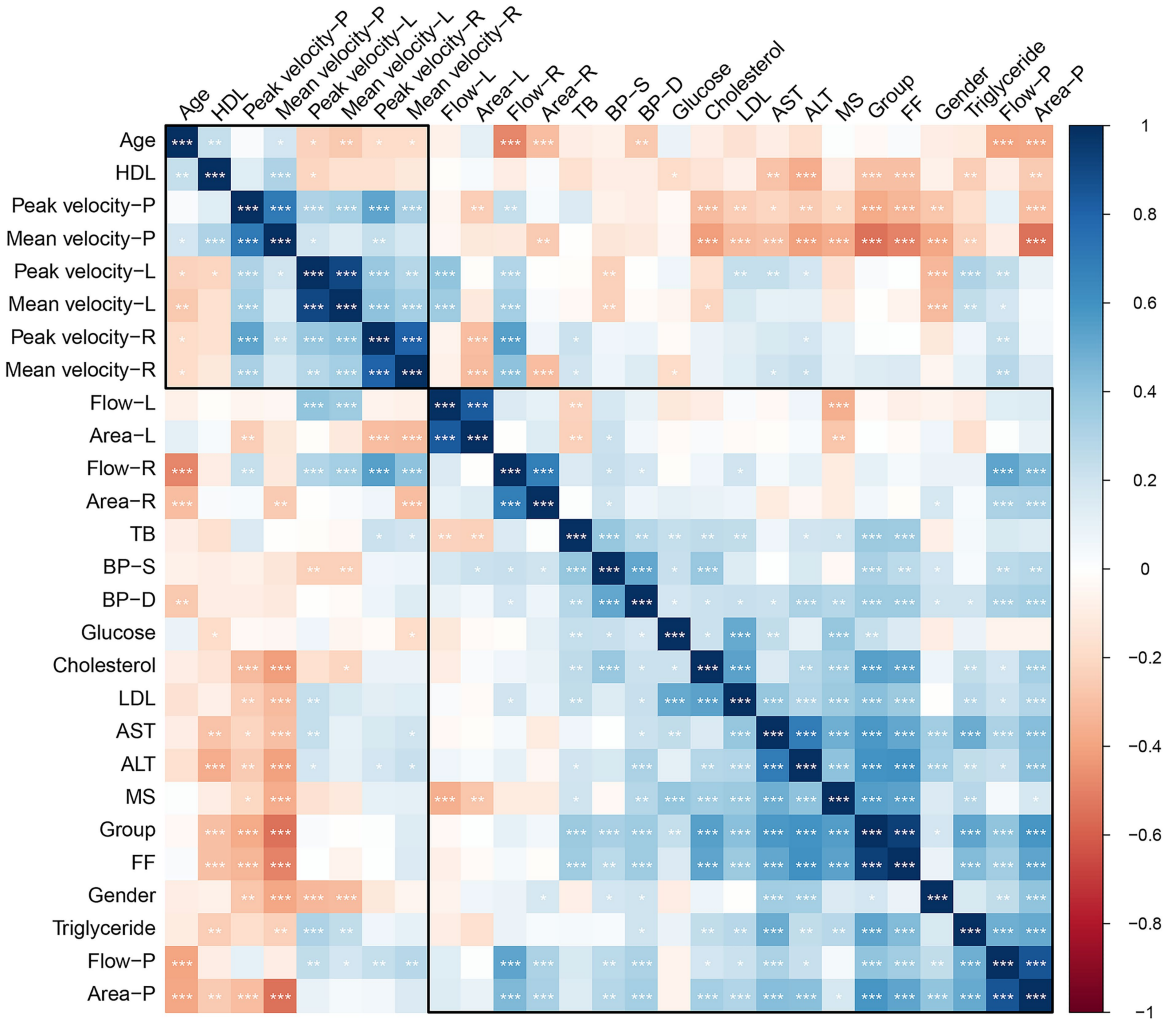
Correlation heatmap of the relationships between various clinical and hemodynamic parameters. The color scale ranges from dark blue (positive correlation, +1) to dark red (negative correlation, −1), with white/light colors representing little to no correlation (0).

Statistical correlations revealed that liver FF was negatively associated with portal peak flow velocity (*r* = −0.33, *p* < 0.001) and portal mean flow velocity (*r* = −0.49, *p* < 0.001). But the liver FF was positively correlated with portal area (*r* = 0.39, *p* < 0.001). No correlation was found in liver FF and portal flow volume (*p* = 0.114). The relationships between various clinical and hemodynamic parameters is shown in Figure 3.

### Diagnostic performance of MRI 4D Flow parameters in grading NAFLD

The diagnostic performance of MRI 4D Flow parameters in grading NAFLD is shown in Table 2. Portal peak velocity demonstrated diagnostic performance across steatosis categories with AUCs of 0.69 (95% CI: 0.57–0.82) for differentiating G0 from G1-3, 0.70 (95% CI: 0.60–0.79) for G0-1 versus G2-3, and 0.57 (95% CI: 0.44–0.69) for G0-2 versus G3.

**Table 2:** The diagnostic performance of MRI 4D Flow parameters in grading NAFLD.

Parameter		AUC	95%CI	SPE	SEN	NPV	PPV
Peak velocity	G0 vs G1-3	0.69	0.57–0.82	0.57	0.93	0.77	0.85
	G0-1 vs G2-3	0.70	0.60–0.79	0.71	0.73	0.79	0.64
	G0-2 vs G3	0.57	0.44–0.69	0.26	1.00	1.00	0.19
Mean velocity	G0 vs G1-3	0.84	0.76–0.92	0.89	0.79	0.62	0.95
	G0-1 vs G2-3	0.78	0.69–0.86	0.59	0.96	0.96	0.62
	G0-2 vs G3	0.70	0.60–0.79	0.56	0.89	0.97	0.25
Area	G0 vs G1-3	0.79	0.70–0.87	0.69	0.76	0.52	0.86
	G0-1 vs G2-3	0.78	0.70–0.86	0.48	0.92	0.90	0.56
	G0-2 vs G3	0.84	0.76–0.92	0.59	1.00	1.00	0.29

AUC, Area Under the Curve; CI, Confidence Interval; SEN, Sensitivity; SPE, Specificity; NPV, Negative Predictive Value; PPV, Positive Predictive Value; US, Ultrasound.

Portal mean velocity demonstrated diagnostic performance across steatosis categories with AUCs of 0.84 (95% CI: 0.76–0.92) for differentiating G0 from G1-3, 0.78 (95% CI: 0.69–0.86) for G0-1 versus G2-3, and 0.70 (95% CI: 0.60–0.79) for G0-2 versus G3.

Portal area demonstrated diagnostic performance across steatosis categories with AUCs of 0.79 (95% CI: 0.70–0.78) for G0 versus G1-3, 0.78 (95% CI: 0.48–0.92) for G0-1 versus G2-3, and 0.84 (95% CI: 0.76–0.92) for G0-2 versus G3.

## Discussion

This study demonstrates an association between liver FF and portal hemodynamic parameters in patients with NAFLD. The findings indicate that increased hepatic fat accumulation is associated with reduced portal flow velocity and increased portal area, which suggests significant alterations in the vascular physiology of the liver in patients with NAFLD.

The utility of advanced imaging techniques in this study builds on previous work, who emphasized the potential of MRI 4D Flow in non-invasive portal vein assessments (13). Additionally, recent study expanded on this by illustrating the advantages of clinical applications of MRI 4D Flow for dynamic blood flow analysis in the portal system (10). Our findings complement previous work by demonstrating the utility of MRI 4D Flow in detecting portal hemodynamic alterations in NAFLD. While prior studies focused on portal hemodynamics in patients with chronic liver disease (14), our study underscores the importance of these metrics in earlier NAFLD stages. The significant correlations observed between liver FF and portal hemodynamic parameters in NAFLD patients suggest that MRI 4D Flow could serve as early indicators for intervention. Furthermore, previous studies highlighted the role of metabolic factors in altering hepatic and portal vein resistance, which may contribute to changes in portal hemodynamics (15). Our study supports these findings by linking hepatic steatosis directly with portal alterations. As a previous study demonstrated the importance of evaluating liver fat independently of fibrosis for predicting portal vein changes (16).

Similar to prior studies, this research highlights the critical role of portal hemodynamic changes as markers of disease progression in NAFLD. Studies have shown that hepatic steatosis contributes to vascular resistance and increased portal pressure, consistent with our findings on reduced portal flow velocity (17). A study found that decreased blood flow velocity occurs to maintain perfusion despite underlying vascular resistance in steatosis (18). Another study showed that fatty liver is associated with an decreased hepatic blood flow velocity characterized by increased intrahepatic resistances in NAFLD. After therapy, portal blood velocity were significantly increased (19). Similarly, the stratification of steatosis grades in our study reveals that Grade 3 patients exhibited the most pronounced reduction in both peak and mean flow velocity.

Multiple studies report that patients with NAFLD, including both adults and adolescents, tend to have a wider portal diameter compared to healthy controls or obese individuals without NAFLD (20). The portal diameter tends to increase with the severity of liver steatosis and fibrosis. In patients with advanced NAFLD, portal diameter was notably increased (21). NAFLD leads to hepatocellular lipid accumulation, which causing increased shear stress and disruption of microcirculation. Accompanied with endothelial dysfunction and vasoconstrictor hyperreactivity, these factors result in higher intrahepatic vascular resistance, elevated portal pressure, and portal vein dilation. The stratified group comparisons showed that portal area does not consistently increase with each steatosis grade, with the most pronounced increase observed in G3 but less distinct differences between G1 and G2. This nonlinear vascular remodeling occurs alongside seemingly stable portal flow volumes across increasing steatosis grades, reflecting dual compensatory mechanisms that balance flow velocity and vessel caliber. In early NAFLD (G1), mild portal dilation may transiently increase flow volume despite modest reductions in velocity, while progressive steatosis (G2-G3) triggers more substantial structural adaptation where further dilation cannot compensate for increasingly reduced flow velocity, ultimately resulting in normalization of flow volume. This dynamic demonstrates how portal vein diameter adapts to preserve perfusion despite rising intrahepatic resistance and declining velocity. Similar hemodynamic adjustments have been observed in animal and human studies of hepatic steatosis and fibrosis, where portal vein dilation acts as a buffer against impaired flow (7, 16).

There is controversy over the changes in portal flow volume in NAFLD. Our study found that in early (G1) NAFLD patients, the flow of the portal vein and its left and right branches was mildly increased, while as the severity of fatty liver worsened (G2 and G3), the portal flow remained stable. This may be due to the dual factors of portal flow velocity and portal diameter regulating the portal flow rate. Early NAFLD patients mainly experience dilation of the portal diameter, resulting in a mild increase in flow rate. Patients with G2 and G3 experience further reduction in portal flow velocity and dilation of the portal diameter, ultimately resulting in the portal flow rate remaining at a normal level. Similar results have been reported in previous studies, where the portal flow/hepatic arterial flow ratio was mildly elevated in G1-G2 patients, but remained normal in G3 patients, and significantly decreased with fibrosis progression (22). Furthermore, increased cardiac output could also lead to incensed portal blood flow, which is observed in NAFLD (23, 24). As elevated systolic BP were observed only in G1 and G2 cases.

Despite the demonstrated correlations between hepatic fat content and portal hemodynamics, the underlying mechanisms remain incompletely understood. The observed vascular changes may reflect not only structural adaptation to hepatic fat accumulation but also metabolic and inflammatory drivers that influence endothelial function and intrahepatic resistance. Furthermore, although this study focuses on non-invasive imaging biomarkers, it does not yet integrate other clinical dimensions such as histologic progression or patient outcomes. Future research should investigate whether MRI-derived hemodynamic markers can predict fibrosis progression or cardiovascular events, which would enhance their translational utility in personalized risk stratification. A multi-modal diagnostic model that incorporates imaging, biochemical, and clinical factors may offer the most comprehensive approach to managing NAFLD in clinical practice.

This study supports the growing body of evidence that liver FF and portal hemodynamic parameters are valuable diagnostic and prognostic markers in NAFLD. Advanced imaging modalities, such as MRI 4D flow, should be integrated into clinical practice to improve early detection and guide interventions aimed at mitigating vascular complications associated with hepatic steatosis. Despite its strengths, including robust imaging techniques and a well-defined cohort, this study has limitations. Its cross-sectional design precludes causal inference between liver fat accumulation and portal hemodynamic changes. While MRI-PDFF provides accurate fat quantification, the absence of liver biopsy limits the ability to distinguish NAFL from NASH or assess fibrosis. The single-center setting and modest sample size may also affect generalizability. Future longitudinal, multi-center studies with histological validation are needed.

In conclusion, this study supports the potential of portal hemodynamic parameters as diagnostic and staging markers in NAFLD. While MRI PDFF and 4D Flow MRI show promise, they remain investigational and require further prospective validation before integration into routine clinical practice.

## Data Availability

The raw data supporting the conclusions of this article will be made available by the authors, without undue reservation.
